# How Similar are Cohabiting and Married Parents? Second Conception Risks by Union Type in the United States and Across Europe

**DOI:** 10.1007/s10680-014-9320-2

**Published:** 2014-08-07

**Authors:** Brienna Perelli-Harris

**Affiliations:** Reader in Demography, School of Social Sciences, University of Southampton, Bldg 58, Room, 2067, Southampton, SO17 1BJ UK

**Keywords:** Cohabitation, Fertility, Childbearing, Europe, United States, Unions

## Abstract

The increase in births within cohabitation in the United States and across Europe suggests that cohabitation and marriage have become more similar with respect to childbearing. However, little is known about additional childbearing after first birth. Using harmonized union and fertility histories from surveys in 15 countries, this study examines second conception risks leading to a live birth for women who have given birth within a union. Results show that women who continue to cohabit after birth have significantly lower second conception risks than married women in all countries except those in Eastern Europe, even when controlling for union duration, union dissolution, age at first birth, and education. Pooled models indicate that differences in the second conception risks by union type between Eastern and Western Europe are significant. Pooled models including an indicator for the diffusion of cohabitation show that when first births within cohabitation are rare, cohabiting women have significantly lower second conception risks than married women. As first births within cohabitation increase, differences in second conception risks for cohabiting and married women narrow. But as the percent increases further, the differentials increase again, suggesting that cohabitation and marriage are not becoming equivalent settings for additional childbearing. However, I also find that in all countries except Estonia, women who marry after first birth have second conception risks similar to couples married at first birth, indicating that the sequence of marriage and childbearing does not matter to fertility as much as the act of marrying itself.

## Introduction

The increasing percent of births within cohabitation across almost all of Europe and the United States indicates that cohabitation is becoming more common as a setting for childbearing (Kiernan 2004; Perelli-Harris et al. 2010, 2012, Kennedy and Bumpass 2008). Family researchers have posited that having children within cohabitation is a sign that cohabitation has taken on many of the functions of marriage (Smock 2000; Seltzer 2000; Raley 2001), reducing the salience of the institution of marriage (Cherlin 2004). However, it is still unclear how similar these two types of unions are when they involve continued childbearing. Although the increase in first births within cohabitation suggests that cohabitation and marriage may be becoming more similar, cohabitors’ second birth risks may differ substantially from those of married couples indicating that fundamental differences remain between the two types of unions. On the other hand, couples may marry after a first birth, suggesting that marriage is not eschewed altogether, but simply postponed until later in the life course (Perelli-Harris et al. 2012). Couples who marry after first birth may be very similar to those who marry before first birth and have similar second birth risks.

This study investigates how second birth risks differ between married and cohabiting parents across Europe and in the United States. Previous studies have focused on union status at entrance into parenthood (Baizán et al. 2003, 2004; Le Goff 2002), the variation in first births within cohabitation (Perelli-Harris et al. 2012), and the correlates of having a birth within cohabitation (Musick 2007; Perelli-Harris et al. 2010; Manning 1993). These studies have provided important insights into how union type shapes the process of becoming parents, and the selection effects associated with having a birth within cohabitation. However, few studies have examined what happens after the first birth and to what extent cohabiting and married couples are similar in having additional children. Given the increase in first births within cohabitation across Europe, this issue is important for understanding whether cohabitation is becoming a long-term setting for childbearing and rearing, at least in some countries. While this study cannot tell us about *all* cohabiting unions, or selection into having a first birth within cohabitation, it provides an important piece of the puzzle on how similar cohabitation and marriage are with respect to childbearing.

To explain any differences between the behaviors of cohabiting and married parents, the study investigates a number of factors. One of the primary reasons for any differences in second birth risks may be union instability; in most countries, cohabiting unions have higher dissolution risks, even if they involve childbearing (Heuveline et al. 2003). To account for differentials in union stability, I control for union dissolution and examine whether cohabiting couples that stay together have different second birth risks than married couples. Because of the interest in testing whether cohabiting and married parents behave similarly, I only examine the unions in which a first birth occurs and do not follow respondents after union dissolution or into a new partnership. Studies have also found that the behaviors of cohabiting and married couples become more similar as union duration increases (Lyngstad et al. 2011; Wiik et al. 2009). Thus, I also include union duration at first birth to account for increasing commitment and union stability over time.

The study also specifically examines second birth risks for cohabiting couples who marry after the first birth. Those who marry after first birth may have similar birth rates to those married at first birth; only the sequence of marriage and birth may be reversed. Also, given that many family formation events have increasingly been postponed (Billari and Liefbroer 2010), some couples may postpone both childbearing and marriage until late in the woman’s reproductive ages, and then have additional children quickly to account for the postponement. Hence, the reversal of the sequence of marriage and birth may be the result of the postponement of marriage rather than couples rejecting it altogether.

Besides investigating second birth risks by union type within countries, it is important to explore differentials across countries. The countries included are available in a database called the Harmonized Histories, which comprises standardized union and fertility histories from surveys around Europe and the United States (see www.nonmarital.org). Together, the countries cover a substantial proportion of Europe’s population, including North–South family patterns described by Reher (1998) and East–West household formation regimes described by Hajnal (Coale 1992). In addition, the database includes the United States, which is purported to be an outlier in family behavior, especially nonmarital childbearing (Cherlin 2009). Below, I discuss the factors that underlie the spread of cohabitation in each country and propose country-specific hypotheses for how second birth risks may differ by union type. By pooling the data, I can test whether any differences between countries are significant. In addition, I test whether the diffusion of cohabitation is related to second birth differentials by union type. As first births within cohabitation increase, cohabiting and married women may become more similar, suggesting that cohabitation is becoming less selective. On the other hand, the relationship may not be linear, but instead reflect a U-shaped pattern of diffusion, as has been found in other studies of cohabitation (Liefbroer and Dourleijn 2006). A U-shaped pattern would indicate that second birth differentials by union type may be different as cohabitation starts to diffuse, become more similar as childbearing within cohabitation becomes practiced by the majority, and then finally become different again as cohabitation becomes the norm. Thus, by pooling the data and including diffusion indicators, we can better understand cross-national variation in second births by union type.

## Theoretical Framework

### Comparisons between Cohabitation and Marriage

As cohabitation has increased, researchers have asked to what extent cohabitation is “indistinguishable from marriage” or an “alternative to marriage” (Heuveline and Timberlake 2004; Kiernan 2004). Childbearing within cohabitation has been one of the fundamental indicators of whether a relationship has become more marriage like (Manning 1993; Raley 2001; Musick 2007; Kiernan 2004). Most studies on childbearing within cohabitation focus on all births (e.g., Kiernan 2004; Heuveline and Timberlake 2004; Heuveline et al. 2003), first births (e.g., Le Goff 2002; Kiernan 2004; Perelli-Harris et al. 2010, 2012) conceptions (e.g., Raley 2001; Manning 2004), or even contraceptive use (Sweeney 2010). These studies find that with respect to reproductive behavior, cohabitation is taking on some of the form and function of marriage. For example, research on the U.S. and Europe has found that premarital pregnancy to single women increasingly prompts transitions into cohabitation rather than marriage, suggesting that cohabitation has become more similar to marriage (Raley 2001; Perelli-Harris et al. 2012).

Nonetheless, little is known about childbearing behavior after a first birth within a cohabiting union, especially in Europe where cohabiting unions are expected to be more similar to marriage. Although partnership is often included as a control variable in models of second birth, few studies distinguish between cohabitation and marriage. Often cohabitation and marriage are combined into one partnership variable (e.g., Bartus et al. 2013; Begall and Mills 2012), or cohabiting individuals are included as unmarried (e.g., Köppen 2010; Kravdal 2007). In most studies, differences found between cohabiting and married individuals are hardly discussed, because it is not the main focus of the study. Studies that explicitly report differences between cohabiting and married couples show mixed results: in Denmark, for example, cohabitors had lower second birth risks than married couples (Gerster et al. 2007), while in Estonia, cohabiting women had a slightly higher likelihood of having a second birth (Klesment and Puur 2010). However, because union status is not the main focus of these studies, the authors do not interpret the results.

The studies that explicitly examine what happens after birth tend to focus on the union and examine whether cohabiting unions are more likely to dissolve or convert to marriage (Lichter et al. 2006; Wu and Musick 2008; Steele et al. 2005; Perelli-Harris et al. 2012; Carlson et al. 2004; Manning 2004). Perelli-Harris et al. (2012) find that in most countries of Europe, few couples marry in the first three years after birth, suggesting that cohabiting couples that remain within cohabitation before and after birth do not rush to marry when they have young children. Also, cohabiting unions in the UK that involve conception or birth are often stronger than those that do not, resulting in long-term committed unions less prone to union dissolution (Steele et al. 2005b).

Thus, as assumed in many studies, it could be that having a child within cohabitation cements a relationship to such a degree that the type of union no longer matters. After all, once a child is born, cohabiting and married couples are similar in many ways: two parents live together and are available to care for the child, maintain the household, and contribute to financial resources (Musick 2007). They have a shared interest in the well-being of their child and may stay together in order to raise the child in a stable household. In European countries, cohabiting fathers have the same rights to raise, care, and make decisions about their children as married fathers. Unmarried fathers are able to establish paternity and gain joint custody over their children, although they may face greater bureaucratic obstacles when doing so (Perelli-Harris and Sanchez Gassen 2012). In addition, many of the social taboos of having unmarried childbearing are also disappearing; surveys from around Europe and the United States point to greater acceptance of childbearing outside of marriage (Kiernan 2004; Thornton and Young-DeMarco 2001). Thus, marriage is increasingly becoming irrelevant to parenting a child in a cohabiting union. As the institutional context of raising children within cohabiting unions becomes more equal, the behaviors of cohabitors and married couples may become more similar. This leads us to expect:

#### **H1a**

**Cohabiting and Married Women have Similar Second Birth Risks**

On the other hand, most studies show that cohabitors and married people are quite different. Individuals who have ever cohabited typically have less traditional family-oriented attitudes, as argued by proponents of the Second Demographic Transition explanation (Lesthaeghe and Neidert 2006; Lesthaeghe 2010). The Second Demographic Transition posits that innovators in new family behaviors, such as childbearing within cohabitation, value self-actualization and autonomy, values that emphasize the individual and not the traditional family unit (Lesthaeghe and Neidert 2006). These values may manifest themselves in individual-oriented behavior, for example keeping economic resources separate, a behavior more prevalent among cohabitors (Lyngstad et al. 2011; Heimdal and Houseknecht 2003). In general, because cohabitors may be less focused on children, they may be more likely to have only one child in favor of other opportunities.

Cohabiting couples may also have less stable relationships, with lower commitment to each other. Studies show that cross-nationally, cohabitors have higher risks of dissolution than married couples (Liefbroer and Dourleijn 2006; Kiernan 2004), leading to higher levels of single-mother families (Heuveline et al. 2003). Studies in the U.S. show that cohabiting women are more likely than married women to be unhappy or dissatisfied with their current situation (Brown 2000, 2003), and cohabiting women suffer higher rates of physical violence and emotional abuse (DeMaris 2001; Kenney and McLanahan 2006). In most European countries, cohabitors have lower levels of subjective well-being (Soons and Kalmijn 2009). Even in Norway and Sweden, where cohabitation is often considered indistinguishable from marriage, cohabiting couples are less serious and satisfied with their relationship than married couples, although this differs for couples with plans to marry (Wiik et al. 2009). Thus, even though cohabiting couples may have one child together, their relationship may be too precarious for them to want more.

In addition, several studies show that lower educated women are more likely to have first births within cohabitation than higher educated women suggesting that childbearing within cohabitation is associated with a pattern of disadvantage (Perelli-Harris and Gerber 2011; Perelli-Harris et al. 2010; Kennedy and Bumpass 2008). Lower education often results in lower wages, less job security, temporary employment, or unemployment. This economic uncertainty may make additional childbearing less affordable or even risky. As a result, cohabiting women in more uncertain situations with fewer social and economic resources could be reluctant to have additional children (note, however, that the fertility of less advantaged groups could also be higher—see below for country-specific hypotheses). Taken together, cohabitors’ unstable unions, lower relationship commitment and quality, and socio-economic disadvantage lead us to the opposite hypothesis of that proposed above:

#### **H1b**

**Cohabiting women have significantly lower second birth risks than married women**

### Delayed Marriage

Although couples may be cohabiting at the time of birth, they may not be rejecting marriage altogether, but instead postponing marriage. In committed relationships, marriage and childbearing may have been jointly planned, with childbearing simply occurring first (Wu and Musick 2008). Just as previous research has found that relationship satisfaction was similar between cohabiting couples with plans to marry and married couples (Wiik et al. 2009), the behavior of cohabiting couples who marry after birth could be very similar to those married at birth. This leads us to predict an additional hypothesis, which is not mutually exclusive from those above:

#### **H2**

**Cohabitors who Marry After First Birth have Similar Second Birth Risks to Those Married at First Birth**

### Variation in Childbearing within Cohabitation Across Europe and the United States

Although the percent of births within cohabitation has increased in all of the selected countries, the variation across countries remains striking (see Table 1). The reasons for this variation are complex and include an interplay between cultural norms, values, and economic factors (Heuveline and Timberlake 2004; Perelli-Harris et al. 2012). Policies and laws governing cohabitation vary considerably across Europe and may guide choices between marriage and cohabitation (Perelli-Harris and Sanchez Gassen 2012). In general, countries adopt new ideas and values at different rates, leading to differentials in family behavior across countries (Lesthaeghe 2010). This variation in family behavior leads to the expectation that Hypotheses 1a and 1b will differ by context (Hypothesis 2 applies to all contexts).

**Table 1:** Percent of first and second births by union status, women aged 15–49, for first births occurring 1985–2000

	Birth 1				Birth 2			
	Cohabiting	Married	Single	*N*	Cohabiting	Married	Single	*N*
Austria	31	54	15	998	22	72	6	797
Belgium	22	68	10	651	19	72	9	488
Bulgaria	12	80	8	2,130	12	82	6	1,291
Estonia	29	57	13	1,093	28	66	6	726
France	37	53	9	1,228	28	66	6	947
Italy	5	90	5	3,539	2	96	2	2,369
Lithuania	8	80	12	1,201	9	87	4	715
Netherlands	14	77	9	1,183	10	85	5	973
Norway	46	43	11	1,796	33	62	5	1,528
Poland	6	80	14	1,541	5	91	4	1,137
Romania	10	86	4	1,339	9	89	3	767
Russia	14	74	12	1,653	11	83	6	758
Spain	12	79	9	1,548	10	87	3	1,124
UK	18	64	18	1,243	16	77	7	955
USA	18	56	26	2,064	16	69	15	1,732

*Sources*: Generations and Gender Surveys in Austria (2008–2009), Belgium (2008–2010), Bulgaria (2004), Estonia (2004–2005), France (2005), Italy (2003), Lithuania (2006), Norway (2007–2008), Romania (2005), and Russia (2004); Fertility and Family Survey in the Netherlands (2003); British Household Panel Survey for the United Kingdom (2005–2006); Poland Employment, Family, and Education Survey (2006); Spanish Fertility Survey (2006); U.S. National Survey of Family Growth (1995, 2006–2008). Weights applied where available

Northern European countries, which exhibit “weak family ties,” tend to have early home-leaving and high levels of cohabitation (Billari and Liefbroer 2010; Reher 1998). These countries are often held up as forerunners of the second demographic transition (Lesthaeghe 2010) and may be experiencing a disassociation between marriage and childbearing. In Norway, included in the study, nearly half of all first births occur in cohabitation, and long-term cohabiting unions are accepted as a setting in which to raise children (Wiik et al. 2009). Cohabitation has become normative (Syltevik 2010), to such an extent that only 10 % of unions in which children are born start with marriage (Perelli-Harris et al. 2012). Policies related to cohabitation are very similar to those of marriage, with fathers given automatic joint custody and few differences in rights and obligations for couples with children (Perelli-Harris and Sanchez Gassen 2012). Therefore, the expectation is that in Norway, cohabiting couples who have a first birth together have similar second birth risks to married couples (H1a).

Although Estonia was part of the Soviet Union and dominated by state socialism, its family formation patterns are similar to Nordic patterns, with high levels of cohabitation that began to increase before the collapse of the Soviet Union (Katus et al. 2007). These union formation patterns may have been the result of an early trend toward secularism, potentially tied to a history of Protestantism. In general, Estonians have liberal values with respect to gender equality and individual freedom of choice. However, Soviet policies in the late 1980s may have prompted cohabiting couples to quickly register their marriages in order to gain access to housing. Thus, unlike in the Scandinavian countries, cohabitation in Estonia in the late 1980s and early 1990s tended to be premarital with less long-term cohabitation (Katus et al. 2007). This leads to the expectation that many cohabiting women will marry after having a first birth. Those remaining in cohabitation may be more selective with lower relationship quality or fewer resources, as discussed above. Thus, I expect that cohabitors in Estonia will have lower second birth rates than those married at first birth (H1b).

France also has a relatively high percent of couples cohabiting: according to this data, about 37 % of first births in 1985–2000 occurred in cohabiting unions. France started to experience an increase in cohabitation in the 1980s, and by 1995–99, more than 90 % of first unions had started with cohabitation (Köppen 2010). According to Martin and Théry (2001), the birth of a child is no longer seen as a sufficient reason to get married. They argue that in long-term cohabiting unions, behavior and values are no different than for contemporary married couples, and even more similar for parents, where rights and duties are exactly the same. Hence, in France, I would expect no significant differences in second birth rates between cohabiting and married women (H1a).

German-speaking countries and the low countries of Belgium and the Netherlands have been slower to experience increases in cohabitation, especially long-term cohabitation that includes childbearing. In Austria, cohabitation has been high in certain regions, but overall, the levels of cohabitation have remained moderate. Although the state has provided generous benefits to single mothers, the state partially favors the breadwinner model and reserves many legal rights for married couples (Perelli-Harris and Sanchez Gassen 2012). Therefore, even though childbearing within cohabitation is increasing in Austria, marriage is generally the preferred situation for raising children. In the Netherlands, although 78 % of women who had a first birth in 1995–2003 started their unions as cohabitation, only 33 % conceived within cohabitation, suggesting that nearly half of all couples marry before first conception (Perelli-Harris et al. 2012). While cohabitation may be increasing as a prelude to marriage, it is not practiced as a long-term relationship for childbearing. In Belgium, the prevalence of cohabitation and childbearing within cohabitation is starting to increase, but nonetheless the decline in marriage may not be that pronounced (Neels 2006). Evidence suggests that marriage is still highly valued (Corijn 2005, 2011), and that while people may be postponing marriage, they do not eschew it altogether (Corijn 2005). Therefore, Belgium still maintains a general orientation toward marriage, especially when involving childbearing (De Wachter forthcoming). Due to the preference for marriage in Belgium, the Netherlands, and Austria, women who have a first birth in cohabitation could have different characteristics than those who marry beforehand. For example, previous research has found a negative educational gradient for first birth rates in cohabitation in Austria and the Netherlands, suggesting that cohabitation is associated with disadvantage (Perelli-Harris et al. 2010). Hence, the expectation is that cohabiting women will have significantly lower second birth rates than married women (H1b).

Southern European countries continue to maintain “strong family ties” (Reher 1998) and conservative values that encourage traditional patterns, such as marriage and raising children within marriage. These values are reflected in the low percent of first births in cohabitation: our sample data show that 12 % of first births in Spain and 5 % of first births in Italy occurred to cohabiting mothers in 1985–2000. In Spain, the mean age at marriage is one of the oldest in the world; yet, Spain has been slow to experience a parallel increase in cohabitation, with couples more likely to “Live Apart Together” (Castro-Martín et al. 2008). This behavior suggests that cohabitation has not been widely accepted as a setting for childrearing. In Italy, the Catholic Church has remained very influential for family formation behavior, although the exact pathways of influence remain ambiguous (De Rose et al. 2008). Some studies show that parents have been “blocking” the uptake of cohabitation in younger generations (Di Giulio and Rosina 2007), implying that cohabiting couples are under pressure to marry, especially after having had a child. Therefore, those that remain unmarried are unconventional, either rejecting the institution of marriage or unwilling to marry for other reasons. All in all, the emphasis on marriage in Italy and Spain leads to the expectation of significantly lower second birth risks for cohabiting women compared to married women (H1b).

Poland and Lithuania are former socialist countries with strong traditional and religious values (Katus et al. 2007; Mynarska and Bernardi 2007). As in other ex-socialist countries, state policies previously required that couples be married in order to obtain access to housing. With the collapse of socialism, these policies were abolished, but the emphasis on marriage continued due to the strong influence of the Catholic Church. In Poland, marriage, particularly religious marriage, continues to be “deeply internalized” (Mynarska and Bernardi 2007). While Poles express general tolerance for cohabitation, and up to one-third of all unions started with cohabitation in 2004–2006 (Matysiak 2009), they are less likely to stay in cohabitation for long periods of time, especially after having a child. Lithuanians have also maintained conservative gender roles and support for the breadwinner model (Katus et al. 2007). These traditional values are reflected in relatively stable trends in direct marriage and only a limited uptake of cohabitation (Katus et al. 2007). Therefore, as in Italy and Spain, I expect that cohabiting couples will differ from married couples and have lower second birth risks (H1b).

Eastern Europe has had a distinct cultural pattern of family formation, characterized by nearly universal, early marriage (Coale 1992). After the collapse of socialism, the age at marriage and level of cohabitation began to increase slowly, but at different rates across the region (Philipov 2007; Hoem et al. 2009). Historically, Russia, Bulgaria, and Romania were influenced by historical kinship patterns that promoted early marriage. During the socialist period, pro-natalist policies encouraged marriage by providing housing to married couples. Despite the strong marriage tradition, however, it is unclear how second birth risks differ by union status, since other factors may influence birth rates. After the collapse of socialism, second birth risks declined dramatically in Russia, Bulgaria, and Romania making second births uncommon (Philipov and Jasilioniene 2007; Muresan et al. 2008). Very low birth rates for all women may reduce the differentials in second birth risks by union type, rendering any differences insignificant. In addition, studies have indicated that cohabiting couples in the region are disadvantaged (Perelli-Harris and Gerber 2011) and on the margins of society, for example members of the Roma population (Koytcheva and Philipov 2008; Muresan et al. 2008). Contrary to the majority of the population, which limits fertility in times of economic uncertainty, these populations may have higher fertility due to the lack of contraception and a general sense of anomie. Indeed, studies from the region show that women with less education have higher second birth risks (Billingsley 2011; Koytcheva and Philipov 2008). Therefore, the low second birth risks of the married population coupled with the higher second birth risk of the disadvantaged unmarried population may be cancelling out any differences between cohabiting and married women. In the Eastern European countries in this study—Bulgaria, Romania, and Russia—I expect no significant differences in second birth rates between cohabiting and married women (H1a).

Finally, some have argued that English-speaking countries have a different pattern of family formation from that of continental Europe. The United States stands out with relatively high levels of divorce, short-term cohabiting relationships, and a high proportion of single mothers (Cherlin 2009, Kennedy and Bumpass 2008). The U.K. is considered similar to the U.S., due to high levels of teenage childbearing and lone mothers, especially among the least educated (Sigle-Rushton 2008). Overall, cohabitating unions in the U.K. are not usually long-term relationships and have become more unstable over time (Beaujouan and Ni Bhrolchain 2011). These trends suggest that cohabitation in the U.S. and the U.K. is not conducive to additional childbearing. Therefore, the fragility of cohabiting relationships in these countries leads to the prediction that cohabiting couples have lower birth risks than married couples (H1b).

### Diffusion of Cohabitation

Although the relationship between cohabitation and marriage may be country-specific and rooted in socio-economic or cultural influences, the relationship may simply be due to the diffusion of cohabitation: as cohabitation increases, differences between marriage and cohabitation decrease. Some studies have found that differences between cohabitation and marriage disappear as the level of cohabitation increases. For example, the higher the level of cohabitation in a country, the lower the gap between the subjective well-being of married and cohabiting couples (Soons and Kalmijn 2009). This result may be due to selection effects: as cohabitation becomes the norm, it could become less selective of certain characteristics, and the differences between cohabitation and marriage could decline. Such an effect could be occurring with respect to second birth risks. As the level of childbearing within cohabitation increases, cohabitors could be more likely to adopt the fertility levels and patterns of those who practice normative behavior—in other words, married people. Therefore,

#### **H3a**

**Differences in Second Birth Risks between Marriage and Cohabitation Disappear as the Percent of First Births in Cohabitation Increases**

A non-significant result, however, may not necessarily indicate that the relationship is non-existent; instead, the relationship between level of cohabitation and second birth differentials may be non-linear. For example, second births risks may differ for cohabiting and married women at one end of the distribution, but also differ at the other end of the distribution, thereby cancelling out the effects. Liefbroer and Dourleijn (2006) found this type of relationship for premarital cohabitation and divorce. They found that when premarital cohabitation was rare, those who premaritally cohabited had much higher divorce rates than those who directly married. As premarital cohabitation became more common, the differences between premarital and direct marriage decreased. But when nearly everyone practiced premarital cohabitation and direct marriage became rare, the differentials widened again. The authors argued that as premarital cohabitation became the norm, direct marriage became selective of couples who had more conservative or traditional family values, perhaps because their religion held taboos against living together without being married.

Such a diffusion effect could occur with respect to second birth risks as well. As the level of childbearing within cohabitation increases, cohabitors could be more likely to adopt the fertility levels and patterns of those who practice normative behavior—in other words, married people. This means that cohabitors would be more likely to achieve the ideal family size in a given country, usually around 2 children per couple (Testa 2012). Therefore, I would expect that as the level of first births within cohabitation increases, second birth risks would be less likely to differ by union type. However, as childbearing within cohabitation become normative and childbearing within marriage becomes rarer, I would expect second birth differentials to increase again. In the current study, I cannot test this exact diffusion hypothesis, because marital births have not yet become rare. Nonetheless, the level of first births within cohabitation may still impact the relationship between marriage and cohabitation in a non-linear manner.

#### **H3b**

**The Percent of First Births in Cohabitation has a Non-Linear Effect on Second Birth Risk Differentials between Cohabitation and Marriage**

## Data and Methods

### The Data

To compare second birth risks across countries, I employ retrospective union and fertility histories from 15 surveys that have been standardized in a dataset called the Harmonized Histories (Perelli-Harris et al. 2009; and see www.nonmarital.org). The data for Austria, Belgium, Bulgaria, Estonia, France, Italy, Lithuania, Norway, Romania, and Russia come from the Generations and Gender Surveys (GGS), which interviewed nationally representative samples of the resident population in each country. Because the GGS is not available for all countries (or the retrospective histories were not adequate for our purposes), I also relied on other data sources. The Dutch data come from the 2003 Fertility and Family Survey (FFS). The data for the UK are from the British Household Panel Survey (BHPS), including the panels from 1991 to 2006. The Spanish data come from the survey of fertility and values conducted in 2006, and the Polish data are from the employment, family, and education survey conducted in 2006. The U.S. data are from the national survey of family growth, conducted between 2006 and 2008. Note that each survey suffers from specific limitations, such as biased response rates or missing data (for details see Perelli-Harris et al. 2010). Nonetheless, validation studies of the basic fertility measures show that the GGS surveys generally reflect official statistics, especially for the most recent periods (Vergauwen et al. 2012).

The Harmonized Histories data include month of children’s birth, entrance into cohabiting union, marriage, and union dissolution. Despite slightly different survey designs, information on births and union formation is relatively comparable. Questions about cohabitation generally refer to co-resident relationships with an intimate partner, which last more than 3 months. In the Italian, German, and Austrian surveys, there is no minimum duration. Registered unions, or PACS, are recorded in the French GGS, but because less than one percent of unions are PACS, they are included with marriages. Although retrospective data have been found to be subject to recall error, especially for the date of entrance or exit from cohabitation and the existence of short-term unions (Teitler et al. 2006), marriage, and birth dates should be more accurate, thereby helping to order the events of interest. Because not all surveys include complete male union histories, I restrict the analyses to women. The focus is on women who gave birth to a first child in 1985–2000 in order to ensure the greatest comparability; some surveys (U.S., Poland, Austria) only interviewed respondents up to age 44 or 49, which limits our ability to test change over time. Second births can occur any time after the first birth to the date of the interview depending on survey (between 2003 and 2008).

### Analyses

In order to test the proposed hypotheses, I conduct three sets of analyses. The first set of models compares second conception risks among married and cohabiting couples in each country. The second set of models examines second conception risks by union status net of the effect of union dissolution. The third set of models pools the 15 countries to examine whether cross-national differentials in the second conception risks by union status can be explained by country-specific factors such as second conception risks or level of childbearing within cohabitation.

The dependent variable for all models is the log-odds of a conception that leads to a second live birth occurring in a given month. As is common practice in fertility studies, I backdate the analyses nine months to the time of conception, in order to capture decision-making processes and avoid changes in union status that may come as a response to a second pregnancy. No information was available on miscarriages or abortions; therefore, all conceptions lead to a live birth. For the first set of analyses, I use discrete-time hazard models for each country separately to estimate the hazard of conceiving a second child. Respondents enter the risk set in the month following their first birth and are censored when they conceive their second child, when they turn 50, in the month and year of interview (which differs by survey), or when their unions dissolve.

In the second set of analyses, I employ competing risk hazard models to examine second conception differentials by union type net of union dissolution. I use a discrete-time framework to estimate multinomial logistic regression using the sample of all person months when respondents were at risk for having a second conception or union dissolution. By defining no event as the reference category, the model is able to estimate the net hazard of either second conception or dissolution.

The final set of models pools all of the country datasets to examine differences in second conception risks across countries. Although it would be useful to try to explain differences through contextual variables such as family policies or cultural attitudes toward cohabitation, these variables are not available for all countries. Therefore, I examine whether differences in fertility patterns or the diffusion of cohabitation can explain the variation. In the first set of pooled models, I include interactions between country and other parameters to account for country-specific patterns. In the second set of pooled models, I estimate the association of the percent of first births within cohabitation on second conception differentials by union type.

### Independent Variables

#### Union Status

The primary variable of interest is the type of union after the birth of the first child. Union status is a time-varying covariate with three possible states: continuously married, continuously cohabiting, and currently married having previously cohabited. Cohabitors who marry after the first birth move from “currently cohabiting” to “married, but previously cohabited.” I also tested transitions from cohabitation to marriage between first conception and first birth, but only three countries had significant results (women who married during pregnancy in Estonia and Romania had higher second conception risks, while in Italy, women who married during pregnancy had lower second conception risks; results available on request). Given the inconsistent results and different theoretical implications for “shot-gun marriages,” the focus here is on changes in union status after first birth.

#### Mother’s Age at First Birth

It is important to control for mother’s age at first birth in all models, because of the implications for the timing of fertility on subsequent fertility and union behavior, and because the age pattern of childbearing differs substantially across the countries in our study. Mother’s age at first birth may impact second birth risks, since women who delay childbearing may compress second births to have them before the end of the reproductive age (Kreyenfeld 2002). On the other hand, early age at first birth is often associated with being in a cohabiting union and increased union dissolution. Mother’s age at first birth differs across countries and over time; for example, in Eastern Europe, mean age at first birth has been much younger than in Western Europe, although recently the age at first birth has increasingly been postponed (Sobotka 2004). Here, I include a linear specification of mother’s age at first birth, although I also tested a quadratic specification.

#### Duration of Union Before First Birth

Previous research has shown that as union duration increases, cohabiting couples become more similar to marital couples, for example in their likelihood to pool economic resources (Lyngstad et al. 2011). In addition, some governments only begin to regulate cohabiting relationships after a certain length of time, such as 2 years (Perelli-Harris and Sanchez Gassen 2012). Therefore, I control for the number of months in the union before the first birth. The expectation is that longer-lasting unions would be more stable, thereby increasing the probability of having additional children.

#### First Birth Cohort

Because fertility risks changed substantially over our period of analysis, I control for the five-year cohort in which a first birth occurred between 1985 and 2000. The reference category is 1985–1989.

#### Duration Since First Birth

This variable is necessary to specify the baseline hazard from first birth to second conception. I tested linear and quadratic specifications of number of months after first birth but found that splines had the best fit. After testing different spline specifications, I included splines that are 13–24 months, 25–36 months, 37–48 months, 49–60 months, 61–72 months, and 72+ months in the individual country models, and 13–36, 37–60, and 61+ in the pooled models. 1–12 months after birth is the reference category in all models.

#### Education

Some studies have shown that women with higher education have higher second birth risks, although part of this is attributable to the time-squeeze effect (Kreyenfeld 2002). Therefore, I include a control variable for the highest level of education achieved in the individual country models. In addition, previous studies have found a negative educational gradient for first births within cohabitation, suggesting that childbearing within cohabitation may be associated with disadvantage (Perelli-Harris et al. 2010; Kennedy and Bumpass 2008). To test for any additional effect of union type by education on second birth risks, I include an interaction between union status and educational level. In the surveys, education is measured at the time of the interview rather than at time of birth. Although this may introduce some biases, due to some women attaining higher education after giving birth, there are relatively few of such cases. I use three simple categories of education (low: less than secondary education; medium: greater than secondary but less than completed university education; and high: university education), which were collapsed based on ISCED classifications included in each survey.

#### Proportion of Cohabitors

As discussed above, the prevalence of cohabitation may change or explain the relationship between union type and second birth risks. To test this, I included a measure of the percent of first births to cohabiting women in a given country and time period. Using information from the harmonized histories, I calculated the percent of births in cohabitation for 5-year cohorts based on the year of the woman’s first birth. This measure was then attached to each respondent based on their birth cohort. I first tested whether the association was linear by interacting the percent of first births with currently cohabiting. I then tested whether the association is a quadratic by following the strategy of Liefbroer and Dourleijn (2006). I included (1) an interaction between percent of first births in cohabitation and currently cohabiting, (2) an interaction between the squared percent of first births in cohabitation and currently cohabiting, and (3) the same interactions for those who changed from cohabiting to married after first birth.

## Results

### Descriptive Results

Table 1 shows the percent and number of first and second births that occurred in cohabitation between 1985 and 2000 in the sample countries. Weights were applied when necessary to show nationally representative results. This table demonstrates that the variation in the percent of first and second births in cohabitation across countries is substantial, with the fewest percent of births in cohabitation in Italy and the Eastern European countries, and the highest in Norway. The table also provides an idea of sample size for second births that occur within cohabitation. The table, however, does not show the probability of women in different unions to have a second birth conditional on having had a first birth in a union. Given issues with censoring, the best way to show these results is to use hazard models that control for compositional effects such as mother’s age and period of first birth.

### Conception Risks by Union Status, Modeled Separately by Country

Discrete-time hazard models of second conception risks allow for Hypotheses 1a and 1b to be tested for each country. Above, I predicted that cohabiting women would have second conception risks significantly lower than married women in all countries (H1b) except for Norway, France, Russia, Bulgaria, and Romania, where cohabiting and married women would have similar risks (H1a). Table 2 presents the odds ratios from hazard analysis for each country separately. The odds ratios indicate that in all countries except Bulgaria, Romania, Russia, and Estonia, continuously cohabiting women have second conception risks that are lower than those of women who were married at the time of the first birth (significant at the .05 level or less). The odds ratios in these 11 countries range from 29 % lower in Spain to 55 % lower in Italy. The results suggest that with respect to childbearing, cohabiting women are different from married women. This is counter to the expectations for Norway and France, where cohabiting and married women were expected to have similar second conception risks. All in all, the consistent difference between married and cohabiting women across countries is striking.

**Table 2:** Odds ratios of second conceptions based on discrete-time hazard models, women aged 15–49 who had a first birth in a union between 1985 and 2000, by Country

	AUS	BEL	BUL	EST	FRA	ITA	LIT	NL
**Union status** (Ref=Married) Cohabit.	0.590*** (−5.01)	0.573*** (−3.33)	0.819 (−1.75)	1.117 (1.06)	0.563*** (−6.93)	0.448*** (−4.20)	0.591* (−1.98)	0.532*** (−5.27)
Married, prev. cohabit.	1.236 (1.65)	0.740 (−1.14)	1.089 (0.48)	1.767** (3.28)	1.141 (0.96)	0.721 (−1.85)	0.640 (−1.06)	1.409 (1.88)
**Education** (Ref=Medium educ.) Higher educ.	1.134 (1.07)	1.497*** (3.43)	0.779*** (−3.30)	0.995 (−0.05)	1.407*** (4.14)	1.230* (2.55)	0.975 (−0.27)	1.313** (2.91)
Lower educ.	1.086 (0.73)	0.909 (−0.73)	2.131*** (8.75)	1.194 (1.15)	1.113 (1.07)	0.916 (−1.87)	1.442 (1.72)	0.729*** (−4.02)
**Birth cohort** (Ref=1985-89) 1990–1994	0.936 (−0.61)	1.065 (0.53)	0.776*** (−3.61)	0.869 (−1.37)	1.088 (0.97)	1.088 (1.66)	0.896 (−1.16)	1.019 (0.23)
1995–1999	1.107 (0.90)	1.300* (2.05)	0.659*** (−5.14)	0.877 (−1.13)	1.263** (2.60)	1.203** (3.20)	0.647*** (−3.99)	1.081 (0.90)
Union duration	0.997 (−1.49)	0.993*** (−3.63)	0.994** (−2.93)	0.989*** (−3.60)	0.994*** (−4.10)	0.992*** (−7.35)	0.985*** (−4.92)	0.998* (−2.23)
Age at first birth	0.965* (−2.33)	0.943*** (−3.61)	0.950*** (−4.57)	0.976 (−1.63)	0.947*** (−5.09)	0.954*** (−7.77)	0.972* (−2.26)	0.958*** (−3.93)
**Months since 1st birth** (Ref=1-12) Duration 13–24	2.431*** (7.69)	2.097*** (5.36)	2.090*** (7.62)	1.224 (1.65)	2.951*** (9.28)	2.510*** (10.97)	1.517*** (3.36)	3.496*** (13.35)
Duration 25–36	1.916*** (4.96)	2.302*** (5.57)	1.881*** (6.09)	0.938 (−0.45)	3.979*** (11.47)	2.873*** (12.45)	1.348* (2.23)	3.624*** (12.16)
Duration 37–48	1.627** (3.27)	1.580* (2.50)	1.495*** (3.48)	1.062 (0.41)	3.185*** (8.45)	3.345*** (14.08)	1.280 (1.71)	2.737*** (7.73)
Duration 49–60	0.992 (−0.04)	1.223 (0.93)	1.468** (3.15)	0.794 (−1.35)	2.632*** (6.15)	3.178*** (12.67)	0.862 (−0.85)	1.400 (1.79)
Duration 61–72	0.910 (−0.45)	0.771 (−0.90)	1.268 (1.75)	0.976 (−0.14)	1.763** (2.83)	2.116*** (6.97)	0.670* (−2.00)	0.886 (−0.47)
Duration 73+	0.339*** (−6.04)	0.129*** (−7.67)	0.442*** (−6.82)	0.285*** (−7.48)	0.530** (−3.21)	1.101 (1.01)	0.276*** (−8.19)	0.145*** (−6.24)
*N* (mo.)	44,501	37,532	145,974	57,595	55,045	218,580	79,435	43,737
χ^2^	218.6	252.1	551.6	136.4	401.0	631.2	237.9	403.3

	NOR	POL	ROM	RUS	SPA	UK	USA
**Union status** (Ref=Married) Cohabit.	0.614*** (−7.30)	0.626* (−2.36)	1.086 (0.47)	0.921 (−0.50)	0.708* (−2.48)	0.554*** (−4.96)	0.537*** (−6.44)
Married, prev. cohabit.	1.110 (1.18)	1.283 (0.94)	1.043 (0.20)	1.153 (0.70)	1.243 (0.91)	1.076 (0.36)	0.886 (−0.99)
**Education** (Ref=Medium educ.) Higher educ.	1.250*** (3.56)	0.745** (−2.79)	0.698* (−2.30)	0.816 (−1.92)	1.535*** (4.23)	1.087 (1.02)	1.231** (2.60)
Lower educ.	0.847 (−1.84)	1.336*** (3.95)	1.371*** (3.64)	1.644* (2.42)	1.044 (0.60)	0.988 (−0.07)	1.338** (2.94)
**Birth cohort** (Ref=1985-89) 1990–1994	1.116 (1.55)	0.962 (−0.39)	0.807* (−2.45)	0.680*** (−4.18)	1.160 (1.89)	0.877 (−1.35)	1.167 (1.48)
1995–1999	1.277*** (3.40)	0.722* (−3.09)	0.740** (−2.95)	0.440*** (−6.39)	1.207* (2.17)	0.945 (−0.56)	1.149 (1.35)
Union duration	0.998 (−1.61)	0.995* (−2.02)	0.993** (−2.74)	0.997 (−1.21)	0.995*** (−3.50)	0.998 (−1.76)	0.993*** (−4.56)
Age at first birth	0.950*** (−6.18)	0.962* (−2.55)	0.941*** (−4.61)	0.943*** (−4.69)	0.964*** (−4.19)	0.958*** (−4.62)	0.992 (−0.79)
**Months since 1st birth **(Ref=1-12) Duration 13–24	3.730*** (14.13)	1.201 (1.86)	1.085 (0.72)	1.288 (1.95)	2.006*** (5.61)	2.225*** (7.67)	1.766*** (6.35)
Duration 25–36	4.631*** (15.62)	1.169 (1.48)	1.081 (0.65)	0.920 (−0.56)	2.303*** (6.65)	2.190*** (6.64)	1.794*** (5.89)
Duration 37–48	3.718*** (11.55)	1.126 (1.03)	0.844 (−1.24)	1.042 (0.28)	2.850*** (8.35)	1.549** (2.99)	1.636*** (4.25)
Duration 49–60	2.840*** (7.71)	0.952 (−0.38)	0.734* (−2.07)	0.868 (−0.85)	2.867*** (8.03)	1.508* (2.36)	1.634*** (3.69)
Duration 61–72	1.901*** (3.82)	0.832 (−1.28)	0.707* (−2.17)	0.855 (−0.89)	2.304*** (5.70)	0.684 (−1.46)	0.983 (−0.09)
Duration 73+	0.614** (−3.04)	0.403*** (−7.50)	0.180*** (−11.85)	0.403*** (−6.43)	0.826 (−1.40)	0.138*** (−6.76)	0.474*** (−4.48)
*N* (mo.)	70,292	78,627	106,617	109,474	100,647	37,937	53,363
χ^2^	551.6	205.7	339.8	182.7	269.5	211.6	198.4

Exponentiated coefficients; *t* statistics in parentheses

^*^ *p* < 0.05, ^**^ *p* < 0.01, ^***^ *p* < 0.001

However, in the Eastern European countries –Bulgaria, Romania, Russia—and Estonia, cohabiting and married women have no significant differences in second conception risks. This confirms the expectations for Bulgaria, Romania, and Russia (H1a), but not for Estonia, where I expected cohabiting women to have lower second conception risks (H1b). In Bulgaria and Russia, conception risks for cohabitors are slightly lower than for married people, while in Estonia and Romania, conception risks are slightly higher for cohabiting couples, but the lack of significance in these countries suggests that the childbearing patterns for married and cohabiting mothers are relatively similar. The lack of difference, however, may be because both groups have very low-fertility risks in general; second conception risks may be so low in these countries that neither type of couple is having children, thus rendering the difference between the two union types negligible.

In all countries except Estonia, women who were in a cohabiting relationship at first birth and married afterward have second conception risks that were not significantly different from women who were continuously married, confirming Hypothesis 2. In Estonia, cohabiting women who married have second birth risks 77 % higher than their continuously married counterparts. Note, however, that in Austria, the Netherlands, Poland, and Spain, the odds ratios are above 1.2, implying that if the results were significant, women who marry after first birth speed up the timing of their second conceptions, relative to their continuously married counterparts. Only in Belgium, Italy, Lithuania, and the U.S. are the odds ratios below one, suggesting that those who marry after birth may have lower second conception risks than those who were married at first birth. In any case, the results do not show strong differences between those who marry before first birth and those who marry afterward, although the lack of significance may be due to small sample size. In general, the similar second conception risks suggest that cohabiting couples who have a first birth and then marry may have similar levels of commitment and ideas about family size to those married at first birth. The findings may also indicate that couples who marry after a first birth planned both events jointly and happened to have a first birth before marriage (Wu and Musick 2008).

Note that these results occur even when controlling for the length of the union in which the first birth occurs, which has been found to be an important distinguishing characteristic of unions in other studies (Lyngstad et al. 2011). Duration of union is a significant variable in 11 out of 15 countries, but it is slightly below one in all countries.^1^ Duration of union acts in conjunction with the other measures of time in the models: mother’s age at first birth^2^ and duration since first birth. The coefficients for these controls are relatively consistent across countries, although the interval between first birth and second conception does differ across countries; for example, Norway seems to have a steep peak of second conceptions between 25 and 36 months after first birth, while Russia has a flat risk of conceptions during the 72 months after first birth. The birth cohort controls also differ considerably, reflecting the fluctuations in second conception risks for different cohorts. For example, we can see how second conception risks in Bulgaria, Poland, Romania, Lithuania, and Russia for births that occurred in the 1990s compared to the late 1980s.^3^

We can also see substantial variation in second conception risks by level of education across our study countries, and again, there appears to be a rough East–West Europe divide. However, an interaction term between education and union type was not significant in any country (results not shown), indicating that the educational differences in second conception rates do not differ by union status. Thus, unlike in other studies, which found a significant educational gradient for first births within cohabitation (Perelli-Harris et al. 2010), education does not play a role in the difference between second conception risks for cohabiting and married couples.

Competing risk hazard models of second conception risks and union dissolution show whether the differentials between cohabitation and marriage hold for those couples whose unions do not dissolve. As discussed above, one of the main reasons for differences in second birth risks may be the higher dissolution risks of cohabiting couples, which would provide less exposure time for being at risk of second conceptions. Overall, I found very few differences in second conception risks (table not shown due to size; available upon request). Most countries had significantly lower second conception risks for cohabiting women compared to married women, and the same four former socialist bloc countries had no significant difference between second conception risks for cohabiting and married couples. The similar results may be because union dissolution directly after first birth is relatively rare: Perelli-Harris et al. (2012) found that <10 % of unions dissolved within three years of a first birth in most countries, although some countries had a much higher percent of unions dissolve than others. The present analysis extends the possible period of observation after birth up to 15 years, but this period may still be insufficient to capture the majority of dissolving unions, especially because the presence of young children may strengthen unions (for example, in Great Britain, Steele et al. 2005b). Therefore, union dissolution does not seem to explain the differences between cohabiting and married women.

### Pooled Models: Differences between Countries

The difference in second birth risks by union type between the Eastern and Western European countries plus the U.S. raises many questions, particularly about the former socialist countries. As discussed above, these countries went through major upheaval after the collapse of the Soviet Union, and their fertility risks plunged to extreme lows, primarily due to the postponement or elimination of second births (Philipov and Jasilionene 2007; Muresan et al. 2008). These countries also experienced a major increase in cohabitation and childbearing within cohabitation (Hoem et al. 2009; Perelli-Harris and Gerber 2011). In order to investigate whether the differences between Eastern and Western countries seen on Table 2 are significant, I pool the Harmonized Histories surveys and run a single-event history model with second conception as the outcome of interest. I include interactions between covariates and country to allow the hazards to vary across countries on all aspects. France is the reference category.

**Fig. 1:**
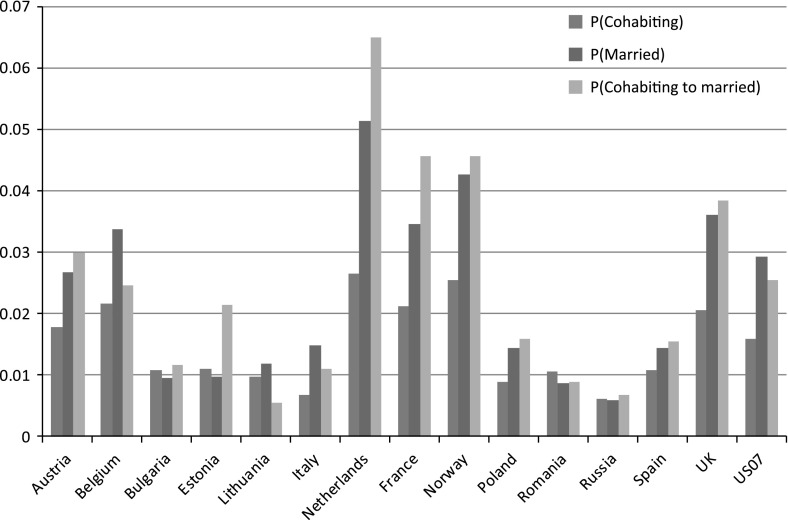
Predicted probabilities of second conception for each union status for women aged 15–49 who had a first birth in a union (estimated 25 years old at first birth, union duration of 31 months before first birth, 13–36 months after first birth, 1990–1995), based on pooled model of 15 countries

Figure 1 presents the predicted probabilities from these models for union status at second conception calculated using the mean age at first birth for all countries (age 25), mean union duration before first birth (31 months), mean duration after first birth 13–36 months, and birth cohort of 1990–1995. Figure 1 shows the range of predicted probabilities of second conception across countries; as expected, the highest probabilities of second conception occur in Norway and the Netherlands, and the lowest occur in very low-fertility countries: Bulgaria, Estonia, Italy, Lithuania, Poland, Romania, and Russia.

Figure 1 also shows that cohabiting women have much lower predicted probabilities of a second conception than married women in most countries, as seen above. However, in Bulgaria, Estonia, Romania, and Russia, the probability of second conception within cohabitation appears to be very similar to that within marriage. An interaction term between country and cohabitation for these countries is significant in the pooled models, indicating that the relationship between cohabitation and marriage is significantly different from that in France (tables available upon request). This result indicates that these countries in Eastern Europe are significantly different from France. Note, however, that the result is not just due to very low second conception risks in cohabitation; Italy and Lithuania also have low cohabiting conception risks, but the interaction term between these countries and cohabitation is not significant, indicating that the relationship between union status and fertility is the same as in France. Thus, the significant interaction term for these countries of Eastern Europe indicates that the association between fertility and union status is significantly different than that in Western Europe, despite the very low second conception risks.

### Pooled Models: Prevalence of Cohabitation

Finally, in order to see whether the diffusion of first births in cohabitation explains the results (H3a and H3b), I ran additional models with the pooled data (Table 3). Column 2 shows odds ratios for a model including the proportion of first births within cohabitation for each country interacted with currently cohabiting. This model tests whether the relationship between cohabitation and marriage changes as the proportion of first births increases. The interaction term for this model was not significant, indicating that Hypothesis 3a cannot be confirmed. However, the lack of significance could be the result of non-linear effects: cohabitation and marriage could be different when cohabitation is rare, become more similar over time, and then diverge again as births within cohabitation become more common. Following the strategy of Liefbroer and Dourleijn (2006), I included (1) the proportion of those cohabiting at first birth interacted with those who were continuously cohabiting (also married after cohabiting at first birth) and (2) the squared proportion of those cohabiting at first birth interacted with those who were continuously cohabiting (also married but previously cohabited). The interaction terms for those continuously cohabiting were significant, but not for those who married after cohabiting (not shown). Note that due to the hierarchical nature of the data, the standard errors may be underestimated. Nonetheless, it is interesting to examine the relative risks of those continuously cohabiting according to the percent who had a first birth in cohabitation.

**Table 3:** Odds ratios from discrete-time hazard models of second conceptions including proportion of respondents cohabiting at first birth, women aged 15−49 who had a first birth in a union between 1985 and 2000, pooled model of 15 countries

	Baseline model	Proportion cohabiting at first birth	Proportion cohabiting at first birth with square
Cohabiting	0.604*** (−6.37)	0.688* (−1.97)	0.299*** (−3.81)
Married, previously cohabiting	1.123 (0.84)	1.121 (0.82)	1.120 (0.81)
Proportion cohabiting at first birth		0.967 (−1.88)	1.043 (1.13)
Proportion cohabiting at first birth, squared			0.999 (−1.58)
Proportion cohabiting at first birth × continuously cohabiting		0.997 (−0.75)	1.050** (3.01)
Proportion cohabiting at first birth squared × continuously cohabiting			0.999*** (−3.42)
*N* (person months)	1,208,486	1,208,486	1,208,486
χ^2^	6,345.2	6,346.0	6,350.7

Exponentiated coefficients; *t* statistics in parentheses

^*^ *p* < 0.05, ^**^ *p* < 0.01, ^***^ *p* < 0.001

Controls include variables from previous model interacted with country. Full model available upon request

**Fig. 2:**
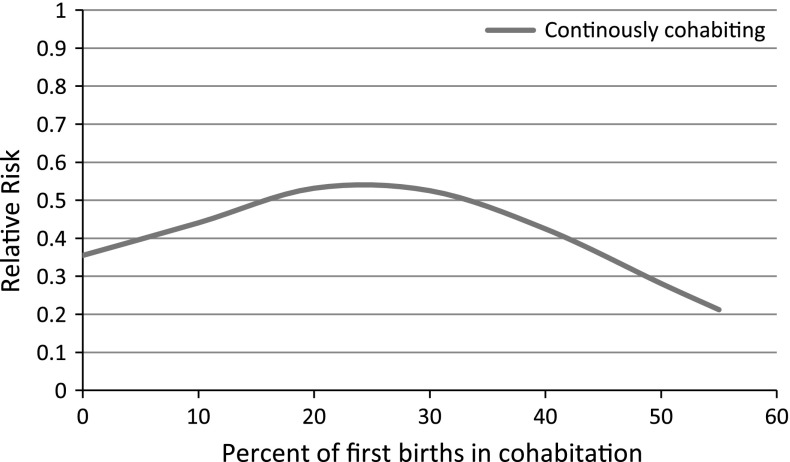
Relative risk of second conceptions for those continuously cohabiting by the percent of first births in cohabitation

Figure 2 shows a U-shaped effect for those continuously cohabiting. The direction of the effect, however, is the opposite of that in the Liefbroer and Dourleijn paper: the U is upside-down. Note that Fig. 2 only shows the relative risks for up to 55 % of first births within cohabitation, which is the maximum percent of first births within cohabitation in the data, and therefore cannot shed light on the relationship as marriage becomes more selective. For this range of effects, second conception risks for continuously cohabiting women start out about two-thirds lower than second conception risks for women married at first birth. As the percent of first births within cohabitation rose to about 25 %, cohabitors had second birth risks about half of those married at first birth. Then, the risk of second conception for cohabitors declined to only about one-fifth of the risk for married women when 55 % of first births were within cohabitation. This indicates that while the difference between cohabiting and married women may narrow as first births within cohabitation start to increase, it widens as first births within cohabitation become more prevalent. Note that these results are not driven by Eastern European countries that have a lower percent of first births within cohabitation and similar second conception risks by union type; the analyses include early cohorts from Western countries such as the Netherlands and the UK (in the UK in 1985, 11 % of first births was in cohabitation, 6 % in 1985 in the Netherlands). Thus, because both country and period are included in the model, the results represent a range of effects from across Europe. Overall, the results suggest that although marriage and cohabitation may become slightly more similar early in the diffusion process, as first births in cohabitation become more normative, cohabitation will not replace marriage as a setting for *additional* childbearing. However, in the long run, the meaning of cohabitation and marriage may change substantially, leading to greater similarity again.

## Discussion

In this study, the findings show that in the U.S. and most countries of Europe, cohabiting mothers with one child have significantly lower second conception risks than married mothers with one child. For these countries, the results are remarkably consistent, with cohabitors having between 40 and 50 % lower second conception risks than married women, even when controlling for duration of union before first birth. The results are also robust when controlling for union disruption; a competing risk analysis distinguishing between unions that dissolved and remained intact shows that cohabitors who stay in a union have lower conception risks than married women who stay in a union. In general, the results suggest that cohabiting mothers are different from married mothers, perhaps due to different fertility preferences as posited by Second Demographic Transition theory (Lesthaeghe 2010), or other constraints, such as poor relationship quality (Brown 2003; Wiik et al. 2009) or lower subjective well-being (Soons and Kalmijn 2009). Selection effects may also play a role: the educational gradient of first births in cohabitation tends to be negative, which may lead to socio-economic disadvantage that discourages additional childbearing (Perelli-Harris et al. 2010). Even though an interaction term between education and union status was not significant for second birth risks, unobserved socio-economic factors may still be responsible for the differentials between cohabitation and marriage.

However, the findings also show that in the former socialist countries of Estonia, Romania, Bulgaria, and Russia, cohabiting and married mothers had no significant differences in second conception risks. Controlling for union dissolution does not change these results; marriage and cohabitation were still not significantly different in these former socialist countries. Our pooled models showed that the Eastern European pattern of second conceptions was distinct from that in Western Europe; second conception risks were very low in the 1990s. The pooled models also showed that the association between union status and fertility in France was significantly different than in Estonia, Bulgaria, Romania, and Russia. This was not the case for all low-fertility countries, including Italy and Lithuania, suggesting that cohabitation and marriage may have different meanings in Western and Eastern Europe, especially regarding childbearing and rearing. In Western Europe, relationship instability, lack of commitment, and socio-economic disadvantage may prohibit additional births. In Eastern Europe, cohabiting women may be disadvantaged or beset by poverty (Perelli-Harris and Gerber 2011), but nonetheless have more births, especially if they are part of a marginalized group (Koytcheva and Philipov 2008; Muresan et al. 2008). Taken together, cohabiting couples’ slightly higher birth risks coupled with the low second birth rates for married women results in similar second conception risks in Eastern Europe.

In all countries, women who cohabited at first birth and then married had no significant differences in second conception risks than continuously married women, except in Estonia, where women who marry after first birth have much higher risks of a second conception. Although the lack of significance may be due to small sample size, in most countries, the risks for those who marry after first birth are higher, suggesting that women who marry speed up the timing of second births. In general, this finding suggests that the most committed couples marry and then conceive at similar or higher risks than those married at first birth. The results are consistent with studies which find that couples with plans to marry have similar relationship satisfaction as already married couples (Wiik et al. 2009). For stable cohabiting couples, the sequence of first birth and marriage does not matter as much as the act of marrying itself.

I also tested to what extent the diffusion of cohabitation could explain any differences across countries and time. The expectation was that cohabitation and marriage would become more similar as the percent of first births within cohabitation increased, as has been found in studies of subjective well-being (Soons and Kalmijn 2009). However, the relationship between the prevalence of cohabitation and cohabitors’ behavior was not linear. Instead, the relationship turned out to be an inverted U. The findings imply that initially, when childbearing within cohabitation was marginal, as in Italy in 2000 or the Netherlands in 1985, cohabiting women had significantly lower second conception risks. As first births within cohabitation increased, the difference in second conception risks for cohabiting and married women narrowed. Then, when more than a quarter of first births occurred within cohabitation, the difference between cohabitation and marriage increased again. When the percent of first births in cohabitation reached its maximum (55 %), second conception rates for cohabitation and marriage were most dissimilar.

The findings point to implications for the meaning of cohabitation which are different than those found in Liefbroer and Dourleijn (2006). Liefbroer and Dourleijn’s U-shaped findings suggest that as premarital cohabitation becomes normative, unions that start with cohabitation and transition to marriage are no longer qualitatively different from direct marriages, although eventually direct marriages do become more stable as they themselves become more selective. The Liefbroer and Dourleijn findings suggest that with the spread of cohabitation, premarital cohabitation does not lead to qualitatively different marriages. However, the findings in this paper show that the diffusion effect is quite different with respect to childbearing. Although cohabitation is increasing as a setting for first births and second birth differentials may temporarily narrow, as cohabitation becomes normative, it is still not becoming a preferred setting for additional childbearing; marriage is still more common for second conceptions. Of course, the meaning of cohabitation and marriage as well as the profile of cohabiting women are changing rapidly, and the shape of the curve may alter again as first births within cohabitation become even more prevalent.

In any case, the relationship between the diffusion of cohabitation and changing behavior is not straightforward. Country-specific explanations, including the cultural, socio-economic, and policy environment, may be better at explaining differences than a simple model of diffusion. In addition, even though countries may have similar outcomes, the reasons underlying the outcomes may differ. For example, although second conception risks may be lower for cohabiting couples than married couples throughout Western Europe, different cultural factors may be operating. The Catholic Church may play a role in countries such as Poland and Lithuania, but in more secular countries like Austria, state policies favoring the breadwinner model may encourage marriage. Therefore, it is important to acknowledge how different cultural, economic, and social forces have produced unique contexts for family formation.

Despite entrenched cultural differences, however, this study shows that in most countries of Western Europe and the United States, cohabiting and married couples do have different fertility behaviors after having had one child together. Second conception risks within cohabitation are much lower, indicating that cohabitation should not be considered “an alternative to marriage” or “indistinguishable from marriage” (Heuveline and Timberlake 2004). We urge researchers, particularly in Western Europe, to recognize this distinction in their models and note that the two types of unions appear to be substantially different, regardless of length of union. On the other hand, cohabitation, childbearing, and marriage are clearly connected, with decisions about each occurring jointly (Wu and Musick 2008; Steele et al. 2005a). Cohabitors can marry and then have behaviors indistinguishable from those who married earlier in the relationship. Therefore, is important to study the interplay between cohabitation and marriage to better understand how these two types of relationship are evolving.

## Footnotes

1 A quadratic for union duration was not significant in any model. A quadratic specification of mother’s age at first birth was significant in 7 models.

2 A quadratic specification of mother’s age at first birth was significant in 7 models and slightly changed the coefficients. In Lithuania, the difference between cohabiting and marital second conception risks was no longer significant, and in the Netherlands, those who were married but previously cohabited had significantly higher second conception risks than those who were married at first birth. However, for parsimony and greatest comparability across countries, models with age at first birth are shown.

3 Models with a time-varying covariate for period since first birth did not differ substantially from the models shown in Table 2. The only change to the main variables of interest occurred in the Netherlands, where women who married after first birth had slightly significantly higher risks of second conception. Tables available from the author on request.
